# Three-dimensional Doppler ultrasound findings in healthy wrist and finger tendon sheaths - can feeding vessels lead to misinterpretation in Doppler-detected tenosynovitis?

**DOI:** 10.1186/s13075-016-0968-3

**Published:** 2016-03-18

**Authors:** Mads Ammitzbøll-Danielsen, Iustina Janta, Søren Torp-Pedersen, Esperanza Naredo, Mikkel Østergaard, Lene Terslev

**Affiliations:** Copenhagen Center for Arthritis Research (COPECARE), Center for Rheumatology and Spine Diseases, Rigshospitalet, University of Copenhagen, Copenhagen, Denmark; Department of Rheumatology, Hospital General Universitario Gregorio Marañón, Madrid, Spain; Department of Radiology, Rigshospitalet-Glostrup, University of Copenhagen, Copenhagen, Denmark; Department of Clinical Medicine, University of Copenhagen, Copenhagen, Denmark

**Keywords:** Ultrasonography, Tenosynovitis, Rheumatoid arthritis

## Abstract

**Background:**

The aim was to investigate the presence of feeding vessels in or in close proximity to extensor and flexor tendon sheaths at the wrists level and in finger flexor tendon sheaths in healthy controls, using 3D ultrasound (US), which may cause pitfalls, in order to ensure correct interpretation of Doppler signals when diagnosing tenosynovitis.

**Method:**

Forty healthy participants (20 women and 20 men age 23-67 years) without prior history of arthritis, tendon diseases or present pain in their hands were included. Twenty participants had 3D Doppler US of the second and third finger and twenty of the right wrist. US was carried out using a GE Logiq E9 unit with a 3D US probe. The colour Doppler settings were to published recommendation.

**Results:**

The feeding vessels in or in close proximity to the tendon sheaths were found in the flexor and extensor tendons sheaths at least once in each participant. No significant difference in feeding vessels was seen between the radial and carpal level in the wrist (p = 0.06) or between the second and third flexor tendon sheath (p = 0.84).

**Conclusion:**

Doppler findings in or in close proximity to the tendon sheaths were common in wrists and fingers in healthy participants. These feeding vessels can be a source of error, not only due to their presence but also because they may be interpreted as being inside the tendon sheath due to blooming and reverberations artefacts. These vessels should be taken into consideration when diagnosing Doppler tenosynovitis.

## Background

Tenosynovitis is frequent in patients with rheumatoid arthritis (RA) [1], and may predict erosive disease [2]. As early and intensive treatment can reduce progressive joint destruction and tendon ruptures, early diagnosis and sensitive monitoring is of great clinical importance. Ultrasonography (US) has been shown to be more sensitive for detecting inflammatory changes in RA compared to clinical evaluation [3]. The use of US for diagnosis and monitoring tenosynovitis was further underlined by the Outcome Measures in Rheumatology (OMERACT) US group, which in 2012 published US definitions for tenosynovitis and proposed a US scoring system [4].

Even though Doppler US is used for diagnosing synovial inflammatory activity in arthritis, it is well-known that Doppler signals may be seen in healthy wrist and finger joints and in relation to entheses [5–8], and therefore it is necessary to distinguish such signals from pathological signals when evaluating joints and entheses. An important part of the OMERACT tenosynovitis scoring system is the Doppler signal both in the tendon sheath and in the tendon, and it is therefore important to obtain knowledge of the normal flow in these areas. Doppler signals from feeding vessels in or in close proximity to tendon sheaths may present pitfalls in evaluation, and there are very limited data available on this topic. In addition, in tenosynovitis some Doppler artefacts, such as reverberation and blooming, can interfere with proper interpretation of pathological flow in the synovial sheath.

Reverberation artefact is when Doppler signals from a vessel are repeated in the lower part of the image, and may cover part of the tendon sheath [9]. Blooming artefact is when the colour reaches beyond the vessel, and this is related to the Doppler gain setting. Blooming artefact may cause vessels in close proximity to the tendon sheath to appear to be inside the tendon sheath.

The vascularization in finger flexor tendons is well-described in anatomical studies [10, 11], and there is specific interest in the vincula vessel system, which passes through the tendon sheath. Studies confirm that there is anatomical variation in the vincula system [12–14], and that the supply of nutrition to the tendons does not occur only at this point. To our knowledge there is only one study describing the vascularization at the wrist [15], and this confirms that there is considerable anatomical variation in the minor arteries.

Three-dimensional (3D) Doppler is a relatively new US technique where a volume is created from a motorized sweep by the transducer inside the transducer housing. When the Doppler is active during the sweep, the vessels will be outlined with colour inside the volume. After the volume has been created it is possible to step through the volume in two dimensions and follow the vessels [16].

3D Doppler has previously been used for the assessment of inflammatory flow primarily in the joints [17–22]. For the present study we chose 3D Doppler over 2D US to better enable the vessels in the volume obtained to be followed, and to better separate the feeding vessels from other vessel in the surrounding area [16]. The aim of this study was to investigate the presence of feeding vessels (seen as Doppler signals) in or close to extensor and flexor tendon sheaths at the wrists and in flexor tendon sheaths at the fingers (second and third) in healthy controls using 3D ultrasound, to highlight possible pitfalls when diagnosing and evaluating tenosynovitis.

## Methods

Healthy participants were included who had no prior history of arthritis or tendon disease, and who were without pain in their fingers or wrists. We assumed the second and third finger flexor tendons were representative of all fingers. Twenty participants had 3D Doppler US examination of the second and third finger and 20 participants had examination of the right wrist. The study was performed following standards for good clinical practice and was assessed by the Danish National Committee on Health Research (no approval was needed). Written informed consent was obtained from all patients.

The US examination was carried out using a GE (Milwaukee, WI, USA) Logiq E9 unit with a 3D ultrasound probe. Colour Doppler was used as it is more sensitive on this machine for slow flow than power Doppler [23]. The colour Doppler settings were according to published recommendations [9] and with a Doppler frequency of 8.3 MHz, pulse repetition frequency (PRF) of 0.4, wall filter (WF) at 66, gain at 20 db, and colour priority at 100 %. The same Doppler settings were used for all examinations. The sweep range was 29 °.

Predefined transverse probe positions were selected before study initiation. For the fingers the sweeps were made on the palmar side in three positions, with the center of the probe positioned over 1) the metacarpophalangeal (MCP) joint (covering the beginning of the tendon sheath for the second or third flexor tendons of the digit), 2) the proximal interphalangeal (PIP) joint (covering the middle part of the tendon sheath for the second and third flexor tendons of the digit) and 3) the distal interphalangeal joint (DIP) (covering the end of the tendon sheath for the second and third flexor tendons of the digit). For the wrist joint three probe positions were chosen on the dorsal aspect and two on the palmar aspect. For each of the probe positions sweeps were made at two different levels (proximal and distal) to cover the entire length of the tendon sheath. The level of the proximal extensor tendons was defined with the centre of the probe at the level of Lister’s tubercle, and the level of the distal extensor tendons with the centre of the probe over the distal portion of the radiocarpal joint. For the proximal level of the extensor tendons the centre of the probe was placed 1) on the radial side of Lister’s tubercle (covering the abductor *pollicis longus*, extensor *pollicis brevis*, extensor *carpi radialis longus* and *extensor carpi radialis brevis*)¸ 2) on the ulnar side of Lister’s tubercle (covering the extensor *pollicis longus*, extensor *indicis*, extensor *digitorum communis* and extensor *digiti minimi*), and 3) on the ulnar to the ulnar head (covering the extensor *carpi ulnaris*). For the distal level, the location of the middle of the probe was unchanged in the sagittal plane. For the proximal level of the flexor tendons the middle of the probe was placed 1) at the radial end (covering the flexor *carpi radiali*s and flexor *pollicis longus*) and 2) at the mid-portion of the pronator *quadratus* muscle (covering the flexor *digitorum superficialis* and *profundus*). No ulnar position was made because the flexor *carpi ulnaris* does not have a tendon sheath. At the distal level, the location of the middle of the probe was unchanged in the sagittal plane. Two sweeps were made at each position to minimise the risk of missing Doppler signals due to different parts of the cardiac cycle being sampled as the sweep was made. All scans were performed by MA-D and IJ and all examinations were evaluated by MA-D.

**Fig. 1:**
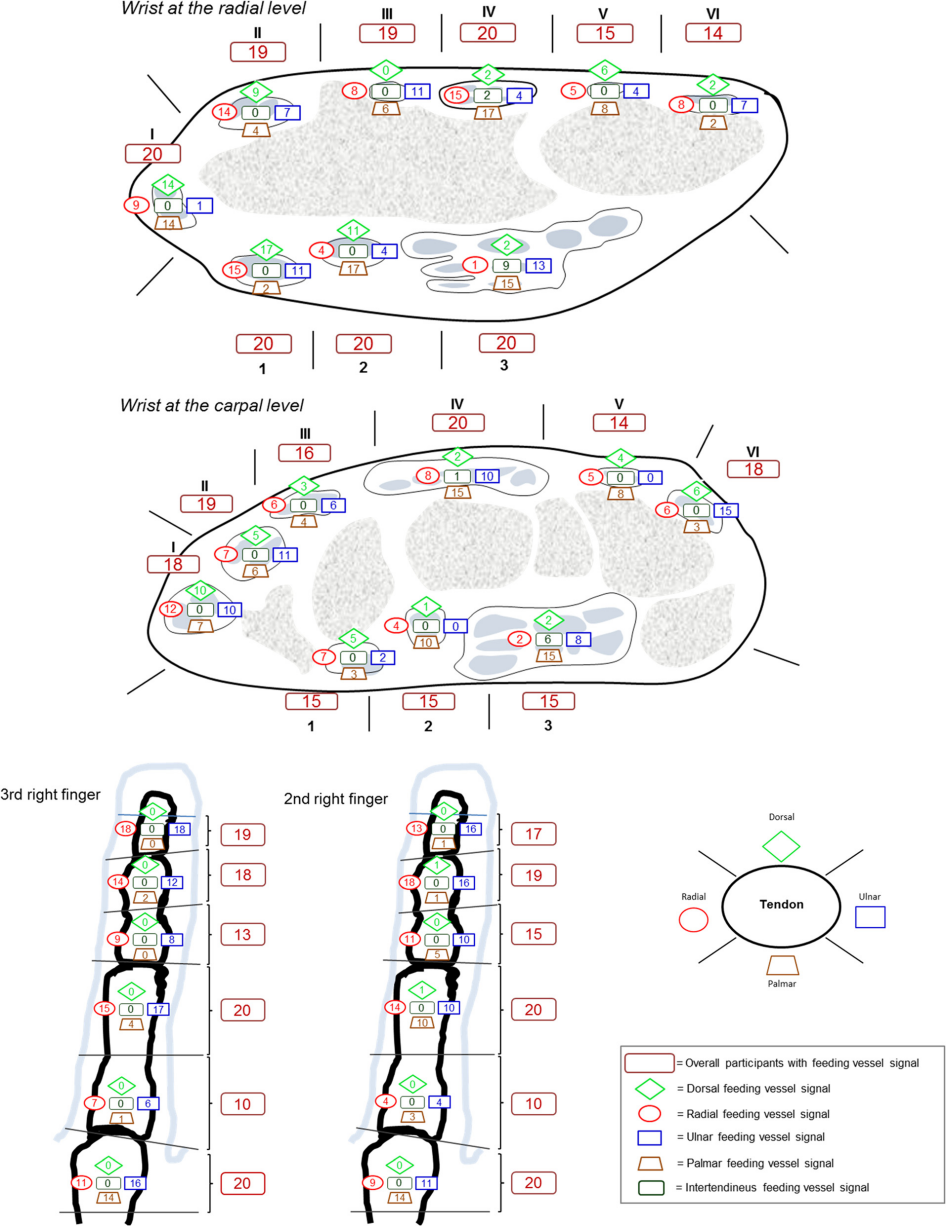
Number of participants with feeding vessel signal at different locations. Distribution of feeding vessel signals in relation to the flexor tendon sheaths of the second and third right finger and the wrist. *Bordeaux boxes* represent the overall number of participants who had feeding vessel signal in the marked area, calculated as presence/absence. *Other boxes* represent the number of participants who had feeding vessel signals at a specific location for the marked area *I* (see *schematic drawing*, *lower right*); abductor *pollicis longus* and extensor *pollicis brevis*, *II*; extensor *carpi radialis longus* and extensor *carpi radialis brevis*, *III*: extensor *pollicis longus*, *VI*; extensor *indicis* and extensor *digitorum communis*, *V*; extensor *digiti minimi*, *IV*; extensor *carpi ulnaris*, *1*; flexor *carpi radialis*, *2*; flexor *pollicis longus*, *3*; flexor *digitorum superficialis* and *profundus*

Each tendon sheath was divided into specific areas (Fig. 1) and 3D Doppler US signals in or in close proximity to the tendon sheath were plotted on a schematic drawing as being either radial, ulnar, dorsal or palmar. The beginning and end (borders) of the tendon sheath was assessed in line with the OMERACT US group recommendations [4]. A feeding vessel was defined as a vessel entering the tendon sheath or aligning with the tendon sheath. The signal had to be seen in the transverse and longitudinal plane over a minimum of 1.8 mm (five frames) and the Doppler signals had to come from a vessel structure (line or well-defined colour dot).

### Statistics

We applied descriptive non-parametric statistical analysis, using SPSS. The Mann–Whitney *U* test was used for comparing the distribution of feeding vessels.

## Results

We recruited 40 healthy participants, 20 women age 25–58 (median 33.5) years and 20 men age 23–64 (median 29.0) years. One of the 40 participants was a smoker. The feeding vessels in or in close proximity to the tendon sheaths were found at least once in each of all the participants.

The distribution of feeding vessels for the wrist is illustrated in Fig. 1. Feeding vessels were common at both the radial and carpal level. Superficial feeding vessels seem to be more common than profound feeding vessels (the feeding vessels closest to the bone) for the extensor tendons, except for the fourth compartment and the first and second compartment at the carpal level. The presence of feeding vessels (calculated per participant as +/- for each compartment (bordeaux boxes in Fig. 1)) for the six dorsal compartments was similar at the radial and carpal level (Mann–Whitney *U* test; *p* = 0.820) with a median involvement of five out of six compartments (25th, 75th percentiles 5.00, 6.00) at both levels. In contrast, feeding vessels in relation to the flexor tendon area were less common at the carpal level than the radial level (*p* = 0.006), with a median presence of feeding vessels in the tendon sheaths of 3.00 (3.00, 3.00) and 2.00 (2.00, 3.00) compartments, at the radial and carpal level, respectively.

Intertendinous feeding vessels were mainly seen in the tendon sheath of the flexor *digitorum superficialis* and *profundus*, but also in the fourth compartment. Intratendinous Doppler signal was seen in the extensor *carpi ulnaris* (ECU) tendon and was only seen twice. No other intratendinous Doppler signals were seen and none of the tendons sheaths showed signs of tenosynovitis.

The distribution of feeding vessels for the second and third finger is illustrated in Fig. 1. The overall segmental distribution (calculated as +/- for each segment) of feeding vessels between the second and third finger did not differ significantly (*p* = 0.84). The median was respectively 5.00 (4.75, 6.00) and 5.00 (4,75, 6.00) for the second and third finger. The feeding vessels were predominantly seen at the ulnar and radial aspects of the tendon sheath, compared to the dorsal and palmar location (*p* <0.0001), except at the distal part of the metacarpal bones, where the palmar location was common. Palmar vessels were more frequent proximally in the metacarpal and proximal phalangeal areas than distally in the intermediate and distal phalangeal areas (*p* <0.0001). Vessels at the dorsal aspect of the flexor tendon were extremely rare, and intertendinous feeding vessels and intratendinous signals were not seen. None of the examined flexor tendons had evidence of tenosynovitis.

## Discussion

**Fig. 2:**
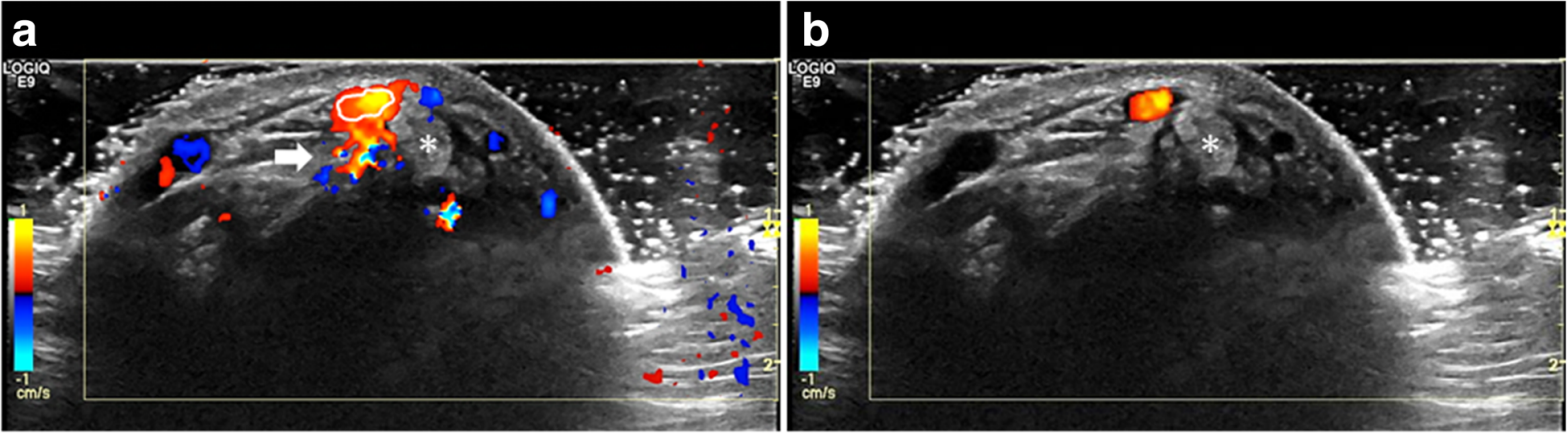
*Compartment 1 (a, b). a Doppler settings were according to published recommendations with optimal Doppler sensitivity. As a consequence of increased sensitivity more vessels were detected but with reverberation artefact (thick arrow) and blooming artefact (the colour immediately outside the white ring) as an unavoidable consequence. b Doppler gain was lowered to avoid blooming. This resulted in loss of Doppler sensitivity and only a single vessel was detected. The outline of this vessel has been traced and the trace is shown on panel a to illustrate the blooming

In this study we demonstrated that feeding vessels in relation to the tendon sheaths are a common finding in the wrists and fingers of healthy participants. None of the examined tendons had evidence of tenosynovitis. These feeding vessels may be a source of misinterpretation, not only due to their presence but also because they cause blooming and reverberations artefacts that may be misinterpreted as perfusion inside the tendon sheath (Fig. 2a and b).

The feeding vessels found in the fingers are in accordance with the anatomical presentation of the vincula system known from anatomical studies [12–14]. Furthermore, our study has shown that feeding vessels proximal to the vincula systems are very common, which to our knowledge has not previously been described in the literature. Whether they have a role in the nutrition of the tendon is unknown and further studies are needed to evaluate this. Feeding vessels in relation to the tendon sheaths in the wrist were also common. Interestingly, intertendinous feeding vessels were seen in the fourth compartment and between the flexor *digitorum superficialis* and *profundus* tendons.

**Fig. 3:**
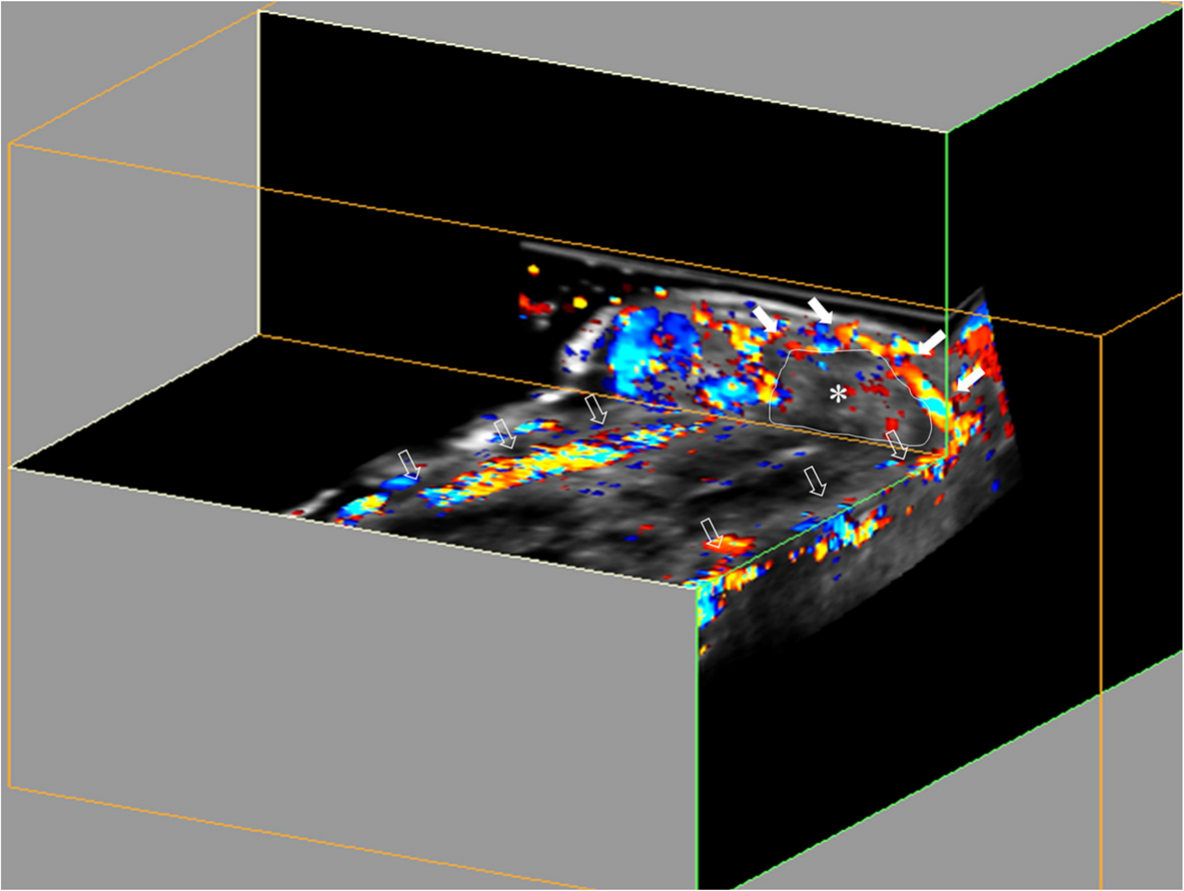
An ultrasound volume video clip of the palmar interphalangeal part of the second finger demonstrates two digital arteries (*open arrows*) and a transverse feeding vessel (*white arrows*). *Flexor tendon and sheath of the second finger. Due to the close proximity to the tendon sheath of the feeding vessel, blooming or reverberation may be misinterpreted, because they seem to be located inside the tendon sheath

**Fig. 4:**
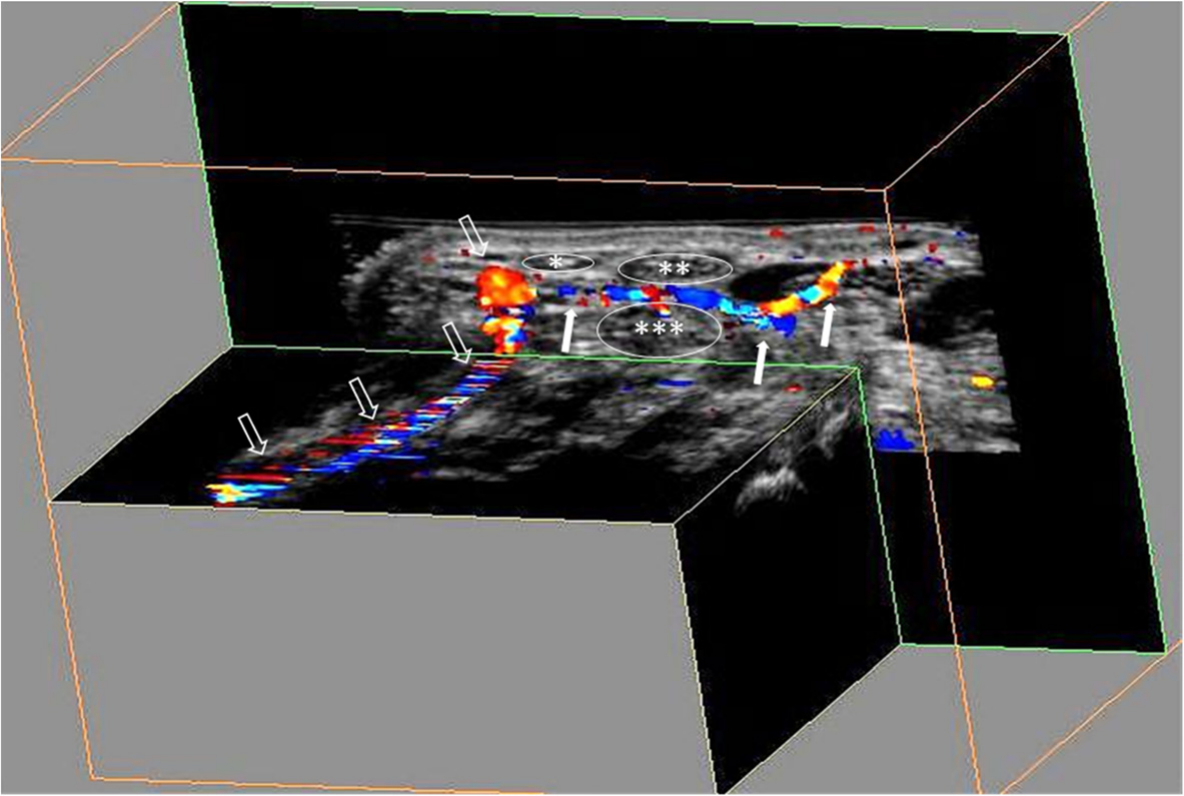
Ultrasound volume video clip of the distal part of the palmar wrist demonstrates the radial artery (open arrows) and a transverse feeding vessel (white arrows). Due to the close proximity to the tendon sheath of the feeding vessel, blooming or reverberation may be misinterpreted because they seem to be located inside the tendon sheath. *Insertion of the flexor carpi radialis; **median nerve; ***one part of the flexor digitorum profundus

The advantage of 3D US examination is that a volume video clip of the whole tendon sheath can be produced, just as with magnetic resonance imaging and computed tomography (Figs. 3 and 4). However, signals from small vessels may be missed by the 3D Doppler scan, as Doppler sensitivity may be lower than for 2D US because of the need for adjustment for different Doppler settings. The potential for lower Doppler sensitivity in 3D US is unavoidable due to the necessity of reducing movement artefacts caused by the movement of the transducer during the sweep. Moreover, if a vessel does not display continuous flow throughout the cardiac cycle it may not be registered by the Doppler US, causing some vessels to be missed when different parts of the cardiac cycle are sampled as the sweep is made. Hence the reason for doing two sweeps per region. With the 2D probe, the Doppler sensitivity is increased but it may be more difficult to follow the vessels and their branches and also to differentiate feeding vessels from muscle perfusion. Above all, our target vessels were physiological feeding vessels, which should not require such high Doppler sensitivity as pathological tenosynovial flow.

It is often reported that the sixth compartment (ECU) is frequently involved in RA [24], and Lillegraven et al. report that tenosynovitis at this location can predict erosive progression in RA patients [2]. Intratendinous Doppler signal in the ECU in healthy participants has not previously been reported, but Doppler signals have been detected in healthy Achilles tendons [25]. Even though the intratendinous signal in ECU was seen in two patients only, the normal results may play an important role when scoring tenosynovitis using the OMERACT US group scoring system, as an intratendinous signal will increase the Doppler grade with one point. The present findings in healthy participants encourage further studies to investigate the implication of these results in a cohort of patients with RA.

.

## Conclusions

Doppler signals in or in close proximity to the tendon sheaths were common in the wrist and fingers in healthy participants. These feeding vessels may be a source of misinterpretation because they may seem to be located inside the tendon sheath, not only due to their location but because of blooming and/or reverberation artefacts. Knowledge of the distribution and specific patterns of Doppler signals in the wrist and fingers are important in order to distinguish pathological from normal findings in the tendon sheaths.

## Abbreviations

DIP: distal interphalangeal joint
ECU: extensor carpi ulnaris
MCP: metacarpophalangeal
MHz: Megahertz
OMERACT: Outcome Measures in Rheumatology
PIP: proximal interphalangeal
PRF: pulse repetitions frequency
RA: rheumatoid arthritis
US: ultrasonography
WF: wall filter
